# Non‐invasive MR imaging techniques for measuring femoral arterial flow in a pediatric and adolescent cohort

**DOI:** 10.14814/phy2.15182

**Published:** 2022-05-25

**Authors:** Jessica E. Caterini, Kate Rendall, Barbara Cifra, Jane E. Schneiderman, Felix Ratjen, Mike Seed, Tammy Rayner, Ruth Weiss, Brian W. McCrindle, Michael D. Noseworthy, Craig A. Williams, Alan R. Barker, Gregory D. Wells

**Affiliations:** ^1^ Translational Medicine The Hospital for Sick Children Toronto Ontario Canada; ^2^ Graduate Department of Exercise Sciences University of Toronto Toronto Ontario Canada; ^3^ Labatt Family Heart Centre Department of Pediatrics Hospital for Sick Children Toronto Ontario Canada; ^4^ Division of Respiratory Medicine The Hospital for Sick Children University of Toronto Toronto Ontario Canada; ^5^ Kinesiology and Physical Education University of Toronto Toronto Ontario Canada; ^6^ Department of Pediatrics The Hospital for Sick Children University of Toronto Toronto Ontario Canada; ^7^ Department of Diagnostic Imaging The Hospital for Sick Children Toronto Ontario Canada; ^8^ Department of Electrical and Computer Engineering McMaster University Hamilton Canada; ^9^ Children’s Health and Exercise Research Centre, Sport and Health Sciences University of Exeter Exeter UK

**Keywords:** cystic fibrosis, exercise, magnetic resonance imaging, test‐retest reliability

## Abstract

Magnetic Resonance Imaging (MRI) is well‐suited for imaging peripheral blood flow due to its non‐invasive nature and excellent spatial resolution. Although MRI is routinely used in adults to assess physiological changes in chronic diseases, there are currently no MRI‐based data quantifying arterial flow in pediatric or adolescent populations during exercise. Therefore the current research sought to document femoral arterial blood flow at rest and following exercise in a pediatric‐adolescent population using phase contrast MRI, and to present test‐retest reliability data for this method. Ten healthy children and adolescents (4 male; mean age 14.8 ± 2.4 years) completed bloodwork and resting and exercise MRI. Baseline images consisted of PC‐MRI of the femoral artery at rest and following a 5 × 30 s of in‐magnet exercise. To evaluate test‐retest reliability, five participants returned for repeat testing. All participants successfully completed exercise testing in the MRI. Baseline flow demonstrated excellent reliability (ICC = 0.93, *p* = 0.006), and peak exercise and delta rest‐peak flow demonstrated good reliability (peak exercise ICC = 0.89, *p* = 0.002, delta rest‐peak ICC = 0.87, *p* = 0.003) between‐visits. All three flow measurements demonstrated excellent reliability when assessed with coefficients of variance (CV’s) (rest: CV = 6.2%; peak exercise: CV = 7.3%; delta rest‐peak: CV = 7.1%). The mean bias was small for femoral arterial flow. There was no significant mean bias between femoral artery flow visits 1 and 2 at peak exercise. There were no correlations between age or height and any of the flow measurements. There were no significant differences between male and female participants for any of the flow measurements. The current study determined that peripheral arterial blood flow in children and adolescents can be evaluated using non‐invasive phase contrast MRI. The MRI‐based techniques that were used in the current study for measuring arterial flow in pediatric and adolescent patients demonstrated acceptable test‐retest reliability both at rest and immediately post‐exercise.

## Graphical abstract

Magnetic Resonance Imaging (MRI) is well‐suited for imaging peripheral blood flow due to its non‐invasive nature and excellent spatial resolution. Although MRI is routinely used in adults to assess physiological changes in chronic diseases, there are currently no MRI‐based data quantifying arterial flow in pediatric populations during exercise. Our objective was to document femoral arterial blood flow at rest and following exercise in a pediatric population using phase contrast MRI, and to present test‐retest reliability data for this method.

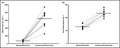

## 1 INTRODUCTION

During exercise, there is a rapid increase in oxygen (O_2_) uptake in the lungs, and elevated blood flow to exercising tissues and O_2_ extraction to meet increased metabolic demand at the contracting muscle (Paterson & Whipp, 1991). O_2_ uptake and volumetric transport to support metabolic processes is termed oxygen consumption (${\dot{V}O}_{2}$), and the peak or maximal value is considered the gold standard measurement of cardiorespiratory fitness and termed peak oxygen uptake (${\dot{V}O}_{2\text{peak}}$) (Harber et al., 2017). Blood flow delivery to tissues plays a key role in determining ${\dot{V}O}_{2\text{peak}}$ (Albouaini et al., 2007; Bassett & Howley, 2000), a predictor of exercise intolerance in many pediatric and adult chronic disease populations (Hebestreit et al., 2005). Evaluating factors limiting exercise capacity is important in chronic disease, and may help identify targets for personalized exercise interventions. Some populations for which this may be important are patients with potential supply/demand limitations to the periphery, such as congenital heart disease (Rhodes et al., 2010) or pulmonary hypertension (Garofano & Barst, 1999). Additionally, this technique may be useful for evaluating exercise limitations in patient populations with vascular abnormalities due secondarily to treatment (patients post‐chemotherapy) (Jang et al., 2013) or primary disease (diabetes) (Naylor et al., 2016).

Fortunately, specific MRI techniques that can interrogate and provide high‐resolution insights at key points in the O_2_ delivery pathway have been developed (Liss et al., 2013; Mathewson et al., 2015; Richardson et al., 2001). MRI is well‐suited for imaging blood flow in the limbs due to its non‐invasive acquisition and excellent spatial resolution. Therefore, the potential sensitivity of vascular flow measurements as a metric of physiological change has resulted in investigation at rest and during exercise using MRI (Klein et al., 2003; Nagaraj et al., 2008). Phase‐contrast MRI (PC‐MRI) has been routinely validated as a method that can provide valuable non‐invasive quantification of blood vessel flow in different adult disease cohorts (Englund et al., 2016; Lalande et al., 2008; Thompson et al., 2016). PC‐MRI measures the area of the vessel of interest and the flow velocity to obtain a volumetric flow rate in a given blood vessel (Klein et al., 2003). Ultrasound is a more accessible measurement modality also frequently used to measure blood flow, but PC‐MRI is less sensitive to errors in vessel area measurement, as it measures each voxel across the vessel lumen simultaneously, and accounts for faster flow in the center of the vessel and slower flow at the vessel wall (Debbich et al., 2020). While PC‐MRI of the peripheral vasculature has been applied in adults, to our knowledge it has not been utilized in a healthy pediatric‐adolescent cohort, which limits its application in pediatric populations both in health and disease. Since pediatric and adolescent blood vessels are smaller than adult blood vessels, this can negatively affect image resolution if an appropriate voxel size is not chosen; therefore, it is important to develop a scalable technique for this unique population (Fukuyama et al., 2017). Fortunately, PC‐MRI applied to fetal circulation in humans and animal models demonstrates its utility in vessels smaller than those of pediatric and adolescent patients, and in nonlinear vessels (Dimasi et al., 2021; Duan et al., 2019; Saini et al., 2020). It is also important to establish reliable, non‐invasive measurement techniques in pediatric and adolescent populations to facilitate research into the pathophysiology of exercise intolerance, which is a hallmark of many pediatric‐adolescent diseases (West et al., 2019). The development of improved techniques such as multiparametric MRI may provide increased acuity in assessing changes in physiology due to exercise stressors, disease progression, or medical treatments in chronic disease.

Therefore, the primary aim of this study was to measure arterial flow in a healthy pediatric and adolescent cohort at rest and in response to exercise using a non‐invasive MRI technique and the secondary aim to assess the test‐retest reliability of this method.

## 2 METHODS

### 2.1 Study design

Research ethics board review was obtained from the Hospital for Sick Children, Toronto, Canada (REB # 1000059989). All participants and/or their parents signed informed assent and consent forms prior to participation. Ten pediatric and adolescent participants (4 male) between the ages of 10–17 years (age median 16 years) were recruited. Inclusion criteria were body mass index (BMI) Z‐scores within −2 ± 2 SD and, normal resting echocardiogram as determined by a pediatric cardiologist (Rodd et al., 2014). Participants were excluded if there was any contraindication to exercise or MR imaging, or if the participant had any known medical conditions for which they were currently being treated. All participants underwent bloodwork (complete blood count) and electrocardiographic and echocardiographic screening for normal cardiac function. Subsequently, participants completed the MRI protocol at rest and during exercise as described below. Participants were requested to abstain from vigorous exercise or caffeine ingestion for 24 h prior to testing. There were no restrictions on food or beverage intake otherwise. Five participants were randomly selected to complete a second visit to assess the measurement reliability, at no less than 2 days and no more than 2 weeks following the first visit.

### 2.2 MRI and exercise protocol overview

All MRI testing was performed on a Siemens 3.0 Tesla PrismaFit imaging system (Siemens Healthcare, Germany) at The Hospital for Sick Children. Baseline imaging of T1 quadriceps structure and phase‐contrast femoral arterial flow preceded all exercise measurements. Exercise was performed using a nonferrous up‐down MR ergometer that was attached to the MRI table (Lode, Germany). The ergometer was set to automatically control power output by adjusting the resistance to the cadence of the participant. Heart rate (HR) was continually recorded during all tests using an MRI‐compatible ECG system.

### 2.3 Exercise protocol

**FIGURE 1:**
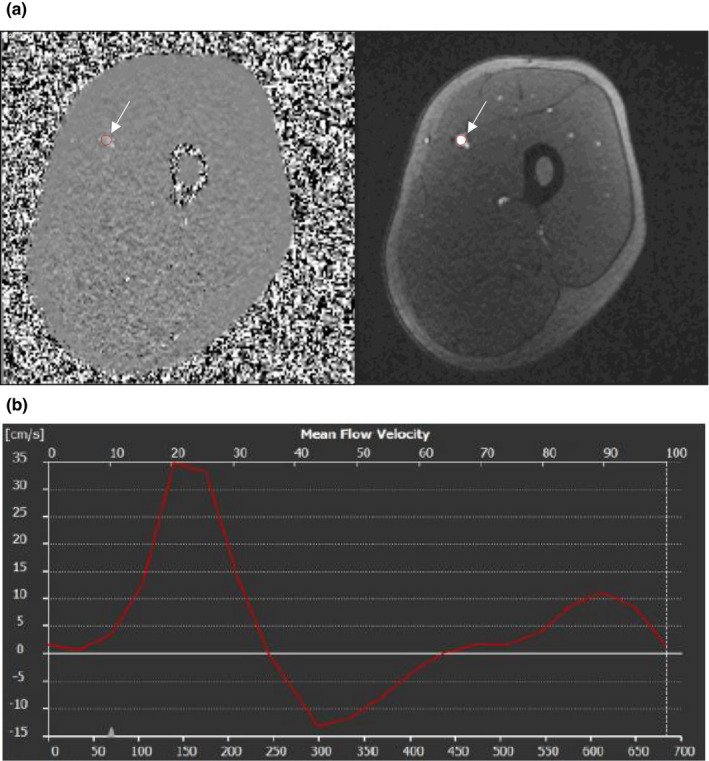
(a) Phase scan (left) and modulus anatomical reference image (right) in a representative participant. The superficial femoral artery is represented by the white arrow and circled in red (QFlow, Medis, Leiden, Netherlands). (b) Sample flow velocity curve obtained from phase‐contrast imaging of the superficial femoral artery at rest in healthy control participant. Heart rate: 88 beats min^−1^; net flow volume 0.50 ml beat^−1^; 44.0 ml min^−1^. Peak flow velocity 75.6 cm s^−1^

Exercise was performed in the MRI as follows: participants had their left quadriceps anchored throughout exercise while they performed a leg kicking motion. Peak power output was determined according to the participant's body mass using previous work in our lab, with maximal power output estimated for weight ranges starting at 12 W for a 40 kg participant, with workload increasing by 2 W every 8 kg (Wells et al., 2011). Exercise began with a 30‐s familiarization interval followed by 5 min of recovery. Participants then performed three bouts of 5 × 30 s single‐leg exercise at 50% of the predicted maximal power output with 15 s of rest in between each 30 s interval (Figure 1a) (Caterini et al., 2015). Participants were encouraged to kick their leg at a steady pace above 10 revolutions per minute, while power output and cadence were monitored and recorded during each test. The mean power output and cadence across the five exercise bouts were recorded. There was a 10‐min break between exercise bouts in the MRI, during which the thigh coil was switched for different imaging methods. Image acquisition was performed as follows: at baseline, which included structural T1‐weighted imaging and during two bouts of exercise that measured PC flow. All structural and baseline images were performed at rest, prior to any exercise. The identical protocol was employed with participants during their second visit with similar morning or afternoon timing, on the identical magnet, with the same resting and exercise sequence order, and identical MR operators to assess test‐retest reliability.

### 2.4 Femoral artery blood flow measurement with PC‐MRI

Femoral artery PC‐flow was collected at baseline during the initial resting sequence prior to any exercise, and immediately following the 2nd 30 s exercise bout. The mid‐point between the knee joint and the hip was selected as the placement for the middle of the coil. Scout images were used to determine artery location and necessary degree of obliquity to ensure a measurement slice perpendicular to the long axis of the femoral artery and vein. T1‐weighted images were acquired to image the arterial lumen as an additional structural guide for flow analyses. Two baseline single slice PC flow scans were acquired at baseline. Subsequently, four PC‐flow measures were acquired immediately post‐exercise, and a single acquisition was acquired 5 min post‐exercise. All flow measures were made through the mid‐point of the quadriceps muscle with the femoral artery as the point of interest as depicted in Figure 1a. The PC MRI acquisition was retrospectively ECG‐gated to permit multiple triggers, and hence temporal measure points, over the cardiac cycle. For example, a typical R‐R interval of approximately 1100 ms resulted in 20 cardiac phases. Images were acquired as follows: velocity‐encoded in the through‐plane direction with matrix size: 256 × 128, 20% phase oversampling; TR/TE = 56.6/3.41 ms, flip angle = 25°, voxel size 1.0 × 1.0 mm, slice thickness = 4 mm, FOV = 200 × 182 mm. Velocity encoding (venc) for baseline measures was set at 100 cm s^−1^, while post‐exercise venc = 200 cm s^−1^, 8 views per 57 segments, with no phase‐wrapping and bandwidth 554 Hz (Lalande et al., 2008). Baseline images were examined immediately following acquisition, and if aliasing was present, the venc was increased to 150 cm s^−1^. The scan time was approximately 19–25 s for each PC‐flow acquisition, depending on the heart rate.

The mean flow velocity was calculated from all scans in each PC acquisition using the commercially available software, QFlow (Medis, Leiden, Netherlands), by a single observer. Flow waveforms were obtained from a tracing of the femoral artery, extracting mean flow volume (ml min^−1^), and mean vessel cross sectional area (mm^2^) for each scan. A sample flow velocity curve is shown in Figure 1b. The average vessel area (mm) was calculated using QMass (Medis). For baseline scans, two image acquisitions were extracted, analyzed, and averaged for each participant.

### 2.5 Statistical analysis

All statistical analyses were performed using Prism 9.1.2 (Graphpad, USA) and SPSS version 22.0 (IBM, USA). The normality of data was assessed using the Shapiro‐Wilks test, and results reported as mean ± standard deviation (SD) or median (interquartile range) if the data were not normally distributed. The intraclass correlation coefficient (ICC), the coefficient of variance (CV), and linear regressions assessed test‐retest reliability for the measurements of resting flow, peak flow and Δ flow rest‐peak on visits 1 and 2. ICC was calculated using a two‐way mixed effects model. Test‐retest reliability, measured with ICC, was determined using thresholds to be excellent (>0.90), good (0.75–0.90), moderate (0.50–0.75), or poor (<0.50) (Koo & Li, 2016). Reliability, measured with CV, was determined to be excellent at values <10% (Sakhare et al., 2019). Between‐day repeatability was assessed with tests of Bland‐Altman mean bias with 95% upper confidence limits (UCL), and lower confidence limits (LCL) reported. Paired T tests were used to determine within‐participant baseline and post‐exercise differences in blood flow and HR. Univariate Pearson or Spearman's correlational analysis was used to determine the relationship between blood flow parameters and participant descriptive variables and hematocrit, and statistical significance was set at *p* < 0.05.

## 3 RESULTS

### 3.1 Participant characteristics

**FIGURE 2:**
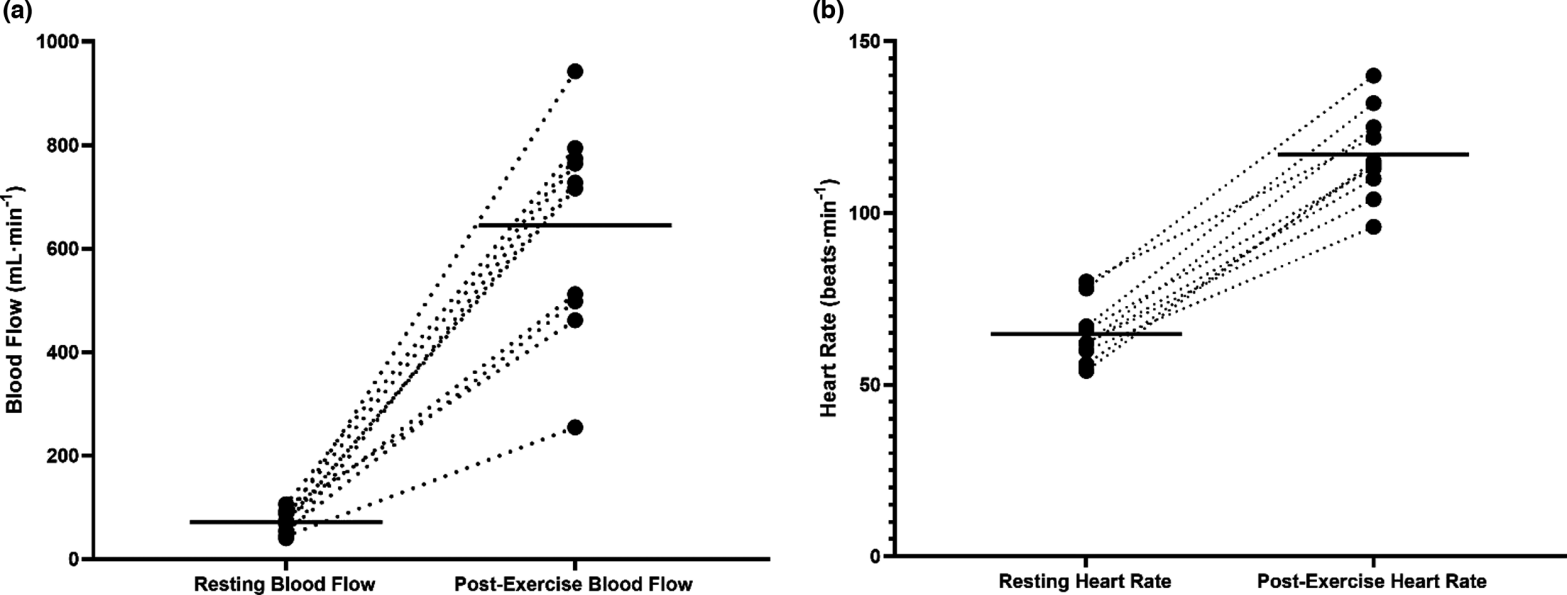
Plot depicting visit one pre‐ and post‐exercise (a) blood flow, and (b) heart rate measurements for *n* = 10 participants

**FIGURE 3:**
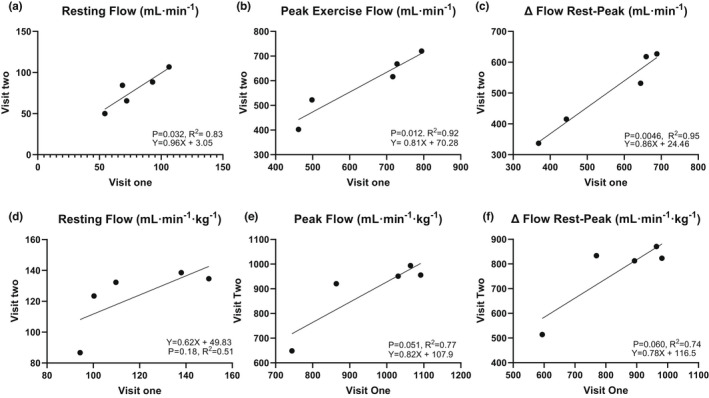
Plots depicting linear regressions between visit one and two (a) resting flow (ml min^−1^), (b) peak exercise flow (ml min^−1^) (c) Δ Flow Rest‐Peak (ml min^−1^), (d) resting flow (ml min^−1^ kg^−1^), (e) peak exercise flow (ml min^−1^ kg^−1^), and (f) Δ Flow Rest‐Peak (ml min^−1^ kg^−1^)

Ten participants completed the study, and five participants returned for a second visit within 2 weeks of initial testing. All participants completed the study. The mean age of participants was 14.8 ± 2.4 years (age range 10–17 years), with a mean height of 161.9 cm (height range 142–177 cm), and a mean hematocrit of 0.39 ± 0.04 (Table 1). Resting HR as measured by MRI plethysmography was 65 ± 9 beats min^−1^, which increased to 117 ± 13 beats min^−1^ immediately at the end of the exercise bout. Peak HR immediately post‐exercise for visits 1 and 2 were not significantly different (Visit 1: 113 ± 8 beats min^−1^; Visit 2 112 ± 11 beats min^−1^, *p* = 0.70) and participants were assigned the same absolute work rate on both visits (Table 2). Pre‐post differences in HR and blood flow are depicted for each participant in Figure 2. All participants successfully adhered to and completed the exercise protocol with no adverse events (Figure 3).

**TABLE 1:** Baseline participant characteristics

	Healthy participants *n* = 10
Age (y)	14.8 ± 2.39
Height (cm)	162 ± 11.8
Weight (kg)	55.1 ± 11.2
BMI	21.0 ± 3.54
BMI z‐score	0.18 ± 1.0
Hct	0.40 ± 0.04
HgB (g l^−1^)	131 ± 18.1
HgB (g ml^−1^)	0.13 ± 0.02
sBP (mmHg)	114 ± 7.22
dBP (mmHg)	65 ± 9
MAP (mmHg)	54.1 ± 5.2

Note

Data reported as mean ± SD.

Abbreviations: BMI, body mass index; dBP, diastolic blood pressure; Hct, hematocrit; HgB, hemoglobin; MAP, mean arterial pressures; BP, systolic blood pressure.

**TABLE 2:** Resting and peak exercise PC‐MRI acquisitions

	All visits pooled (*n* = 10)	Visit one (*n* = 5)	Visit two (*n* = 5)
Resting heart rate (beats min^−1^)	64.7 ± 8.51	61.6 ± 3.58	60.4 ± 5.68
Heart rate during exercise (beats min^−1^)	117 ± 13.0	113 ± 7.66	112 ± 11.3
Power output (W)	7.90 ± 2.10	8.40 ± 1.95	8.40 ± 1.95
Cadence (rev min^−1^)	12.4 ± 6.02	11.8 ± 4.82	12.0 ± 4.85
Resting flow (ml min^−1^)	71.9 ± 21.0	78.9 ± 20.7	79.0 ± 21.9
Resting flow (ml min^−1^ kg^−1^)	120.4 ± 24.8	118.4 ± 24.3	123.1 ± 21.1
Peak exercise flow (ml min^−1^)	645 ± 205	640 ± 149	586 ± 126
Peak exercise flow (ml min^−1^ kg^−1^)	1088 ± 351.8	958.8 ± 149.1	894.0 ± 139.5
Δ Flow rest‐peak (ml min^−1^)	573 ± 199	561 ± 145	506 ± 127^a^
Δ Flow rest‐peak (ml min^−1^ kg^−1^)	968 ± 354.1	840.4 ± 160.9	770.8 ± 145.2
Δ Flow rest‐peak (fold change)	9.48 ± 4.04	8.43 ± 2.26	7.81 ± 2.31

Note

Data reported as mean ± SD. Paired sample T tests were used to compare means between visit one and two.

^a^ *p* < 0.05 difference between visit one and two.

### 3.2 Femoral artery flow

**FIGURE 4:**
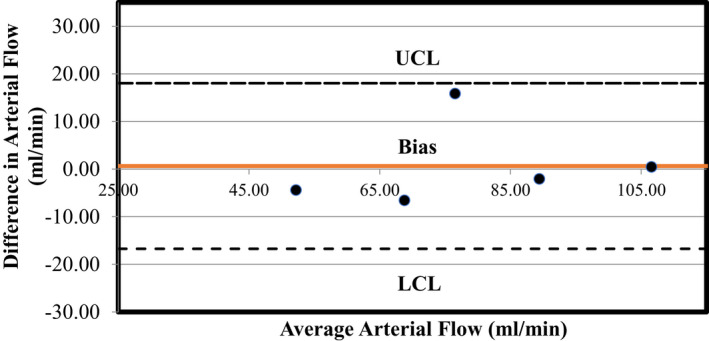
Bland‐Altman plot of test‐retest difference in arterial flow versus average arterial flow for *n* = 5 participants between two visit dates

The mean values for resting and peak exercise femoral artery flow are summarized in Table 2. Mean resting artery area was 21.8 ± 6.5 mm^2^, which is approximately 20 voxels in area for each participant (*n* = 10 participants). Exercise resulted in a significant increase in femoral blood flow. The mean Δ flow rest‐peak fold‐change for visit one was 9.48 ± 4.04. For the five participants who repeated testing, baseline flow demonstrated excellent reliability (ICC = 0.93, *p* = 0.006), and peak exercise and delta rest‐peak flow demonstrated good reliability (peak exercise ICC = 0.89, *p* = 0.002; delta rest‐peak ICC = 0.87, *p* = 0.003) between‐visits. All three flow measurements demonstrated excellent reliability CV values in the five participants who repeated testing (rest: CV = 6.2%; peak exercise: CV = 7.3%; delta rest‐peak: CV = 7.1%). Bland‐Altman statistics for inter‐visit arterial flow are shown in Table 3 and Figure 4. The mean bias was small for femoral arterial flow. There was no significant mean bias between femoral artery flow visits 1 and 2 at peak exercise. Resting hematocrit was positively and moderately correlated with absolute baseline flow (r = 0.75, *p* = 0.012), however, the correlations between hematocrit and peak flow and Δ flow rest‐peak respectively (r = −0.063, *p* = 0.86; r = −0.14, *p* = 0.69) were insignificant weakly and non‐significantly related. There were no significant correlations between age or height and any of the flow measurements. There were no significant differences between male and female participants for any of the flow measurements.

**TABLE 3:** Bland‐Altman coefficients in repeat visit femoral arterial flow

	Bland‐Altman measurement bias				# of measurements within percentage of measurement				
	Mean	*p* value	LCL	UCL	10%		20%		30%
Femoral arterial flow between‐visit reliability									
Flow (ml/min)	0.65	0.88	−16.7	18.04	2		7	9	
Δ flow rest‐peak (ml/min)	−53.8	0.07	−145.1	46.7	2	7		9	
Δ flow rest‐peak (%)	−0.61	0.39	−3.38	2.16	5	7		9	

## 4 DISCUSSION

To the best of our knowledge, we describe for the first time a non‐invasive MRI‐based method for evaluating peripheral arterial blood flow in children and adolescents. We have demonstrated that this method is practical and reliable both at rest and following exercise. We found PC‐MRI to have either good or excellent test‐retest reliability for all blood flow parameters assessed. This method can be used to assess the factors that contribute to exercise intolerance in pediatric‐adolescent cohorts and to evaluate treatment efficacy in clinical trials focusing on these populations. Although delivery of oxygenated blood to peripheral vasculature is not likely to be impaired in a healthy pediatric cohort, it can be reduced by a chronic illness. Moreover, exercise can often accentuate minor differences in blood flow (Wells et al., 2011). Therefore this method could be used to assess peripheral blood flow abnormalities in pediatric and adolescent chronic disease. Identifying impairments in systemic blood flow in response to exercise can help improve overall understanding of exercise pathophysiology in pediatric and adolescent diseases characterized by decreased ${\dot{V}O}_{2\text{peak}}$, especially in cardiorespiratory diseases.

Common techniques traditionally used to evaluate changes in blood flow in the exercising limbs involve non‐invasive methods such as Doppler ultrasound (Maroto et al., 1993; Moalla et al., 2012). However, this method is limited by tissue depth penetration and can have poor spatial resolution, as well as errors in estimation of cross‐sectional area and flow measurement depending on the approach angle, and if the vessel is not circular (Gill, 1985). Near‐infrared spectroscopy can also be used during whole body exercise, but does not measure blood flow per se, and has significant inter‐site variation for oxygenation data (Ferrari et al., 2004). Likewise, these methods do not provide a clear mechanism underlying decrements due to disease or improvements in response to training or treatment. The development of improved techniques to measure blood flow such as multiparametric MRI provides increased acuity in assessing changes in physiology due to exercise stressors, disease progression or medical treatments in chronic disease. We therefore chose to investigate the efficacy of using MRI for this purpose. These novel data characterize pediatric femoral blood flow non‐invasively using MRI at rest and in response to exercise. The exercise protocol used in this study was selected due to its successful implementation among pediatric patients with primary ciliary dyskinesia, CF (Wells et al., 2011), and healthy controls (Banks et al., 2014). This protocol can, therefore, be easily adapted for use in other clinical pediatric populations.

### 4.1 Peripheral blood flow at rest and exercise

Previous studies characterizing peripheral blood flow using non‐invasive MRI techniques have largely focused on adult populations (Banks et al., 2014; Lalande et al., 2008; MacDonald et al., 2000); therefore, all subsequent studies in this discussion will refer to data obtained from healthy adults. There is significant variation in the literature regarding reported volumetric femoral artery flow values. Studies evaluating PC cine imaging of the superficial femoral artery in healthy adults have reported values significantly greater than the ones observed in this study (study mean flow ± SD: 71.9 ± 21.0 ml min^−1^), with values such as 158 ± 74 ml min^−1^ (Englund et al., 2016); and 230 ± 68 ml∙min^−1^ (Alexander et al., 2001). Imaging studies of the common femoral artery (CFA) in adults report absolute volumetric flow that was more than 2–3 times greater than our reported values (Lalande et al., 2008). However, the current study examined the superficial femoral artery following arterial bifurcation, so it is expected that flow will be significantly reduced relative to the CFA. Further, the smaller muscle mass of children and adolescents necessitates a smaller volumetric flow delivery to the peripheral limbs, and blood flow measurements needs to be scaled to reflect the size of muscle (Alexander et al., 2001). Additionally, when we attempted to extrapolate our relative flow values using the absolute volumetric flow data reported by Lalande et al. (2008) the resulting absolute flow values were 2 L·min^−1^ per single‐leg, indicating that the lower limbs were receiving 4 L∙min^−1^ of cardiac output at rest, which does not seem physiologically tenable (Bassett & Howley, 2000). Therefore, there is not only a significant variation between studies in reported flows but also a large standard deviation of reported values. Our results in children and adolescents are slightly lower than reported values, which makes sense given their smaller muscle size. It is also important to obtain pediatric data to make inferences on skeletal muscle physiology rather than extrapolating from adult data, therefore it is difficult to make a direct comparison to existing literature, highlighting the need for research to compare data within a single study.

Size differences between adults and pediatric or adolescent patients may also affect image acquisition. Spatial and temporal resolution with PC‐MRI are important determinants of data quality, and a diameter of four pixels within the vessel lumen is required to obtain accurate PC‐MRI measurements (Jiang et al., 2015). The average vessel cross‐sectional area in our study was approximately 20 mm^2^. This indicates that approximately twenty pixels were included in the vessel lumen with a 1.0 × 1.0mm voxel size, limiting the change of biasing from partial volume effects. Improvements in pulse sequence design and spatial resolution will facilitate our understanding of pediatric‐adolescent hemodynamic responses post‐exercise, with the goal of applying these techniques to study the pathophysiology of exercise intolerance in chronic disease.

At rest, the skeletal muscles receive around 20% of total cardiac output, but during maximal exercise this number increases to 80–90% (Joyner & Casey, 2015). However, these data were obtained from adult populations and studies on animal models, and therefore may not be reflective of the pediatric‐adolescent population. Additionally, exercise stress can help uncover differences in muscle blood flow stemming from underlying pathophysiological processes. The physiological response of femoral blood flow to dynamic exercise using PC‐MRI has not been characterized. We, therefore, quantified the physiological response of peripheral blood flow to a series of 30‐s exercise bouts in the magnet. Post‐exercise blood flow to quadriceps muscle increased nine‐fold over baseline, with a mean HR of 117 ± 13 beats min^−1^. The low heart rates observed in this study can be explained by a small muscle mass being activated and the supine body position during exercise. Other studies using Doppler imaging to quantify femoral flow following leg kicking exercise at 14 W in adults documented similar responses with a 7‐fold increase in flow and a HR of 98 beats min^−1^ (Limberg et al., 2010). Moreover, another study that reported HR increases consistent with those observed in this study demonstrated an almost 11‐fold increase in femoral artery blood flow, as measured by Doppler ultrasound in the final minute of exercise, when HR was 122 beats min^−1^ (MacDonald et al., 2000). Therefore, our exercise regimen evoked significant changes in limb blood flow, which was consistent with other studies in the literature that demonstrated increases in HR comparable to our study.

There are limited data on repeatability of femoral artery measurements using PC‐MRI at rest and during exercise. A single study evaluating the SFA flow repeatability between exercise bouts found a mean difference of 23% in three adult participants for rest‐exercise changes (Nagaraj et al., 2008). Reliability of flow measurements have been evaluated in other large lower limb arteries such as the popliteal artery, with similarly excellent test‐retest repeatability as our study in healthy control patients at rest (ICC = 0.89, CV = 6.8%). Interestingly, patients with chronic arterial disease had significantly higher variability (CV = 15.8%), which suggests that repeatability should be assessed in a pediatric chronic disease population (Versluis et al., 2011) Our present study has excellent reliability for repeat flow assessments at rest (ICC = 0.93), and good reliability for post‐exercise changes in flow (ICC = 0.87), and peak flow (ICC = 0.89). The coefficients of variation for rest, peak exercise, and rest‐exercise change were less than 10%, representing minimal variability between assessments. These reliability values represent acceptable reliability measurements and suggest our PC‐MRI method is robust in a pediatric population. To keep measures as consistent as possible between visits, we asked participants to report back to the MRI at the same time for repeat testing to control for diurnal variation. Additionally, the same MR technologist completed between‐visit scans, the same work‐rate was set for each visit, and heart rate and cadence were carefully monitored to match during each exercise visit.

There are limitations to the current study. All studies using MRI are very expensive, are often constrained by resource availability, and long sequence acquisitions are not suitable for younger children. By nature of their cost, they inherently have smaller sample sizes, of which this study is no exception. Future assessments using this technique should employ a larger sample size and evaluate test‐retest reliability in chronic disease populations, as the low variability we observed in the present study may not apply to a disease group with greater physiological heterogeneity (Versluis et al., 2011) Additionally, any variability in measurements made on the same day on participants in a repeatability study can only be ascribed due to errors in measurement themselves, and we could not control for any biologic variability between visits 1 and 2. Furthermore, the PC‐MRI technique does not directly quantify flow and volume, and represents an interpretation based on sequencing technique.

## 5 CONCLUSIONS

The quantification of peripheral hemodynamics using MRI in a pediatric population is a novel area of research. We demonstrate that non‐invasive imaging is both feasible and has good to excellent reliability in a small sample size of pediatric and adolescent participants both at rest and following and exercise stressor. The non‐invasive nature of MRI makes it an ideal modality for measuring components of limitation in the respiratory chain in response to exercise. Other non‐invasive methods to measure the physiological response to exercise, such as whole‐body ${\dot{V}O}_{2\text{peak}}$, cannot directly pinpoint the limitations in the respiratory chain. These methods may be particularly relevant for patient cohorts that are very small in number, have potential vascular anomalies, or have extremely specialized treatments with limited access to expensive therapies, such as patients with cystic fibrosis, post‐thrombotic syndrome, or congenital heart diseases (Rhodes et al., 2010). The potential for increased study power with advanced imaging techniques is appealing for this population and can approach to disease evaluation and progression.

## CONFLICT OF INTEREST

The authors declare no conflicts of interest.

## AUTHOR CONTRIBUTION

Conceptualization, Caterini, J.E.; Wells, G.D.; Methodology, Caterini, J.E.; Wells, G.D.; Investigation, Caterini, J.E.; Wells, G.D., Cifra B.; Data Curation, Caterini J.E.; Wells, G.D. Writing – Original Draft Preparation, Caterini, J.E., Wells G.D.; Writing – Review & Editing, Caterini J.E.; Rendall K.; Felix, R.; Williams C.; Barker A.; Wells, G.D.; Schneiderman,J.E. Supervision, Wells, G.D.; Project Administration, Wells, G.D., McCrindle B; Funding Acquisition, Wells, G.D.
